# Nursing Education during the SARS-CoV-2 Pandemic: Assessment of Students’ Satisfaction with e-Learning Environment

**DOI:** 10.3390/ijerph19042023

**Published:** 2022-02-11

**Authors:** Emilia Moreno-Sánchez, María-de-los-Ángeles Merino-Godoy, Sara Piñero-Claros, Alba Santiago-Sánchez, Ángela Del-Campo-Jiménez, Laura Mariscal-Pérez, Francisco de Paula Rodríguez-Miranda, Emilia Isabel Costa, Francisco-Javier Gago-Valiente

**Affiliations:** 1 Department of Pedagogy, Faculty of Education, Psychology and Sports Sciences, University of Huelva, 21007 Huelva, Spain; emilia@dedu.uhu.es (E.M.-S.); francisco.paula@dedu.uhu.es (F.d.P.R.-M.); 2 Nursing Department, Faculty of Nursing, University of Huelva, 21007 Huelva, Spain; 3 Nursing Department, Hospital Universitario Puerta del Mar, 11009 Cádiz, Spain; sara.pinero599@alu.uhu.es; 4 Nursing Department, Health Center Cazalla de la Sierra, 41370 Seville, Spain; alba.santiago764@alu.uhu.es; 5 Nursing Department, Hospital Arnau de Vilanova, 25198 Lleida, Spain; angela.delcampo540@alu.uhu.es; 6 Nursing Department, Hospital Juan Ramón Jiménez, 21005 Huelva, Spain; laura.mariscal774@alu.uhu.es; 7 Nursing Department, Health School, University of Algarve, 8000 Faro, Portugal; eicosta@ualg.pt12; 8 Area of Preventive Medicine and Public Health, University of Huelva, 21004 Huelva, Spain; francisco.gago@dstso.uhu.es

**Keywords:** education, COVID-19, virtuality, nursing, public health

## Abstract

The disease caused by the SARS-CoV-2 coronavirus led to the disruption of normality with respect to education, public healthcare and new technologies. Education is a fundamental pillar to increase the knowledge and morale of people. However, due to the lockdown implemented to protect the population from an infection of unknown aetiology, the education system decided to switch from face-to-face education to virtual education. This modality has affected the teaching–learning process in the Degree of Nursing, since its competencies and knowledge demand in-presence learning. The aim of this study was to evaluate the impact that telematic education had on students of the Degree of Nursing who were studying in the final year of said degree, which involves their imminent entry into the labour market. We used the client satisfaction questionnaire of Bob Hayes to gather data and analyse the satisfaction level of the nursing students. As a result, a considerable amount of information was obtained about teaching, which shows the absence of practical activities and the lack of information about safety and protection measures related to the pandemic. Most educators themselves were struggling to understand the implications of the virus and implement appropriate safety measures, since there was quite a bit of conflicting information relating to the effectiveness of personal protective safety equipment and the lifespan of the virus on various media outside of the host. It is, therefore, not surprising that education for students in this regard was lacking. In general, most of the students showed dissatisfaction with the virtual education they received.

## 1. Introduction

This study was carried out during a period marked by an extreme event on a global scale. The pandemic situation has had a profound impact on several key structures in countries across the world, particularly at the level of health systems and, inevitably, on health professionals. This period of time was a consequence of the severe acute respiratory syndrome coronavirus 2 (SARS-CoV-2) that resulted in a worldwide COVID-19 pandemic.

Given the severity of the health situation in March 2020, the Spanish Government declared a state of alarm, which was extended until 21 June, with home confinement to stop the spread of the virus. These events had an important psychological impact on the population, such as fear, pessimistic feelings and irritability [1,2], in addition to other social and psychological problems [2,3]. 

The Spanish economy suffered severely [4], health systems were on the brink of failure and schools were closed. This last aspect jeopardizes the quality and level of education of many children and young people, worldwide, at the various educational levels, thus worrying the United Nations Educational, Scientific and Cultural Organization (UNESCO). These additional consequences increased the seriousness of the impact of the pandemic on the population, since, as is established in the Jakarta Declaration on Leading Health Promotion into the 21st Century (World Health Organization (WHO)) [5], education is a valuable investment, as it accelerates the progress toward a world alliance for the improvement of the quality of life [6].

In this context, the crisis caused by SARS-CoV-2 had a double effect on the nursing profession, transforming it into an occupation of special risk. Healthcare teams have reported the lack of investment to strengthen their professional and specialized duties [7]. “The reports of the World Health Organization (WHO) on the situation of nursing in the world, in addition to stating the need for investment in education, work and leadership in nursing, also reveal the unequal distribution of the nursing staff in the world and within each country, which generates important inequities of professional care for the population” [8].

Regarding teaching, universities hastily adapted their teaching activity to the e-learning environment. The closure of campuses, the interruption of social interaction and the increase in the number of Internet-mediated learning activities had several consequences, such as the significant decrease in the number of activities and physical contact [9]. Among university students, sedentary time increased, and the activities of body and mind relaxation diminished [10]. This contributed to generating psychological discomfort [11], which is reported in studies that describe anxiety, depression and social dysfunction among university students [12,13].

In principle, the remote teaching model in Spain was essentially based on the dissemination of content through virtual platforms and teacher–student asynchronous communication through tools such as e-mail, forums and chats [14]. This suggests that, rather than remote education, this should probably be called “emergency remote teaching” [15], which was achieved thanks to the technological infrastructure that developed countries already had [16].

Students and teachers had to operate in a totally unknown terrain [17]; specifically, in the studies conducted in the scope of health, e.g., with the Degree of Nursing, the adaptation was difficult. Attaining a quality remote education required innovations [18,19], such as the use of educational strategies based on virtual simulation [20,21]. Faculty members had to conceptualize and offer alternative experiences to ensure the provision of an education that would adequately prepare students for professional qualifications in the health field [22].

The present study analyses the satisfaction of fourth-year students (final year) of the Degree of Nursing with the online education received (Supplementary File). It is important to highlight that the attitudes and disposition of university students toward remote teaching has been shown in previous works to be positive [23,24]. However, among nursing students, it has been reported that remote education is not suitable for their degree, that the system does not tackle the competencies that must be acquired and that it is not appropriate to carry out the clinical practical activities in virtual hospitals [25].

The model of focus and evaluation in the current study is categorized as a techno educational model, widely referred to as the Community Inquiry Model (CoI), a model which has been developed and described through hundreds of studies guided by Garrison, Anderson and Archer [26] over the past decade. Participation, motivation and learning/interaction within a globalizing and integrating constructivist perspective form the basis of the model. Theoretically, the model holds that the construction of knowledge in virtual teaching–learning environments (EVEA) takes place through the development of a community, which is characterized by three “presences”: teacher, social and cognitive [27]. The community itself would be defined as a group of people in an educational, cooperative, open, participatory and flexible learning project [28]. More specific aspects of the model related to this research will be detailed in the study procedure.

The aim of this study was to evaluate the satisfaction of fourth-year students of the Degree of Nursing at different universities at the international level (Supplementary File) during the period of e-learning that ensued due to the SARS-CoV-2 pandemic in the academic year 2020/2021. Thus, we analysed the influence of such teaching methodology on nursing students, considering also the influence of sociodemographic variables.

## 2. Materials and Methods

### 2.1. Participants

This is a quantitative, non-experimental, cross-sectional and descriptive investigation. The sample consisted of 400 students in their final year (4th year) of the Degree of Nursing, with an age range of 21 to 52 years, and a mean age of 23.29 years.

Most of the students were from Spanish universities (98.25%), with the participation of many Andalusian faculties (64.5%).

Regarding the distribution as a function of sex, 82.75% were women (*n* = 331) and 17.25% were men (*n* = 69). It must be taken into account that most nurses are women; therefore, in the percentages of samples in studies related to these professionals, the representation of women is always notably higher than that of men. With respect to the marital status, 5.8% had a partner (*n* = 22), 1.8% were married (*n* = 7), 1% were divorced (*n* = 4), 0.3% were widowed (*n* = 1) and 91.1% we single (*n* = 366).

### 2.2. Procedure

The participating students were randomly selected. We decided to choose 4th-year students of the Degree of Nursing based on the fact that this population has intragroup characteristics that make it relevant for this study, such as the realization of professional practical activities and their imminent entry into the labour market.

To calculate the sample size, we estimated the minimum sample required to consider a parameter with a proportion of 50 in a population of unknown size. Taking into account these data, and considering a confidence level of 95%, a precision of 10% and 5% possible losses during the data cleaning process, we calculated that the minimum sample size for this study would be 120 individuals. However, to conduct more complex analyses that involved sample segmentation into subgroups of individuals, it was decided to obtain a sample of around 400 individuals.

The data were gathered through a self-administered questionnaire. The field work was carried out from 10 March 2021 to 23 April 2021. The questionnaire was sent to the participants in a severe period of the SARS-CoV-2 pandemic, where the cases increased continuously, and hard restrictions were enforced.

The methodology of the classes in the universities was carried out through the medium of communication that was established in this period of the pandemic, in which the development of interaction and the participation of all members of the community was encouraged, which gave advantages to the shyest people, fostered the empowerment of the autonomous and self-regulatory character of learning itself and led to the strengthening of collaborative learning [26]. The “social presence” through the virtual platform of the subjects allows us to understand how the participants in virtual teaching–learning environments (EVEA) are projected as “real” people, especially in contexts of asynchronous communication based on texts (forums), that show the affection, group cohesion and communicative openness necessary to establish a sense of trust and belonging to a community oriented to the construction of knowledge. The schedule established for each subject was synchronous. There were diaphanous tasks, such as group work, which were conducted in classes. All classes and tutorials (longer, flexible and personalized) were held on Zoom. The interaction was in classes, in group tutorials and in individual consultations. Positive relationships and the development of training activities with tutorials were encouraged.

Different student delegations of Andalusian universities were contacted. Universities from Galicia, the Canary Islands, the Balearic Islands, Castile and Leon, La Rioja, Castilla-La Mancha, the Community of Madrid, Extremadura, Asturias, Murcia, Navarra, the Community of Valencia, Cantabria, the Basque Country and Catalonia also participated in this study. Furthermore, international students from universities in Portugal, Chile and the USA were also included in the sample. Contact was made formally via e-mail and telephone, and informally through WhatsApp, Facebook and Instagram, to obtain the sample. An information sheet was distributed, in which the students could access the questionnaire with the possibility of translating it to a different language. The format of the instrument was designed using Google Forms, and the data were gathered directly using this platform. 

Written informed consent was obtained from all participants for the analysis of the data and the publication of the results. Anonymity was ensured. In this way, we guaranteed the compliance with the current regulations on the protection of personal data contemplated in Organic Law 3/2018, of December 5th, on Personal Data Protection and Guarantee of Digital Rights. Moreover, the guidelines of the Declaration of Helsinki were respected at all times. The study was approved by the Research Ethics Committee.

### 2.3. Instruments

To gather the data of the main variable of the study, we used the client satisfaction questionnaire of Bob Hayes [29,30,31,32]. A reliability analysis of the instrument was performed, obtaining a reliability of 0.948 (Cronbach’s alpha), which indicates that the degree of reliability of the instrument is acceptable. Moreover, the questionnaire has been validated and widely used in studies with similar populations [33]. It evaluates the satisfaction of the student with online teaching through the following 4 items:

Item 1 (curricular aspects):
  - Adequacy of content for thematic understanding.
  - Level of content detail.
  - Clarity in the presentation of content.
Item 2 (didactic proposal):
  - Usefulness of documents for the understanding of the topic.
  - Adequacy of the amount of study material proposed.
  - Coherence between the proposed activities and the desired material.
  - Coherence of the proposed evaluation with the performed activities.
  - Efficacy of the applications of collaborative work for learning.
Item 3 (technological environment):
  - Ease of access for the different materials.
Item 4 (tutoring):
  - Faculty response time.
  - Clarity and relevance of the messages of the teacher.

A Likert scale was used to assess these 4 items, with 1 being the minimum score (totally dissatisfied) and 5 the maximum score (totally satisfied). If the score in the statement of the item was 3 or lower, it was considered that the student did not show favourable satisfaction in this aspect.

Furthermore, a final item was included, which gathered information about the use of tutoring with the faculty member, allowing the student to choose among the following response options: “Always”, “Generally”, “Sometimes” or “Never”.

Sociodemographic variables were also included, such as sex, age, marital status, province of residence and the university in which they were registered.

The following information was also gathered in this study relative to the impact of the SARS-CoV-2 pandemic on student perceptions and satisfaction: suffering from the disease (COVID-19) caused by this virus, carrying out clinical practical activities during the pandemic and the perception of preparation for the world of work.

### 2.4. Analysis of Data

Statistical analysis was conducted using IBM SPSS Statistics v.22.0 software. The descriptive analysis was carried out using the measures of central tendency (mean and standard deviation) and frequencies and percentages, using frequency tables.

Specifically, for the variable of age we calculated the mean and standard deviation.

We calculated the frequencies and percentages of the following variables: sex, realization of face-to-face practical activities, marital status, university of origin, suffering from COVID-19 and the perception of preparation for the world of work.

To study the possible dependency between student satisfaction in virtual education and the rest of the variables, after verifying the normality of the quantitative variables, we performed the following tests:

- Pearson’s Chi-squared, to analyse the dependency between student satisfaction with virtual teaching and suffering from COVID-19, sex and the realization of face-to-face practical activities.
- Student’s *t*-test, to determine the dependency between student satisfaction with virtual teaching and age.

## 3. Results

### 3.1. Realisation of Face-to-Face Practical Activities and the Perception of Preparation for the World of Work

Of the 400 individuals who participated in the study, 333 (83.3%) performed clinical practical activities during the home confinement period, whereas the other 67 participants could not perform such activities (16.8%). Of these 400 total participants, only 169 (42.3%) claimed to be prepared to face the world of work.

### 3.2. Prevalence of Suffering from COVID-19 among the Participants

**Figure 1:**
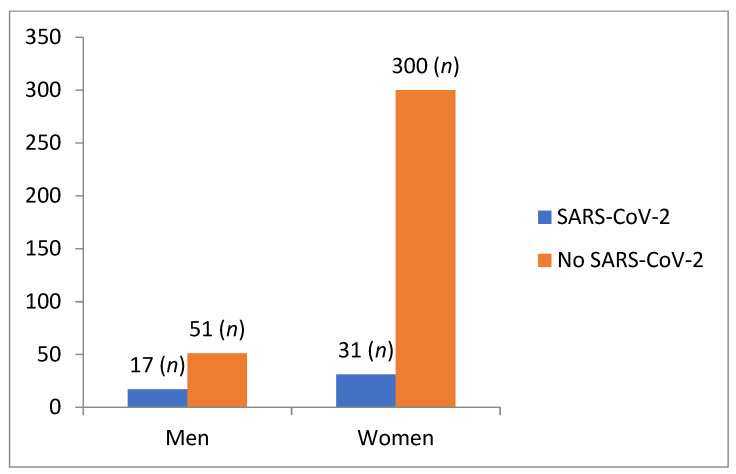
Frequencies of suffering from COVID-19 as a function of sex.

A total of 15.25% (*n* = 61) of the students had suffered from COVID-19 at the time of participating in the study, whereas 84.75% (*n* = 338) did not. Figure 1 represents these data categorized as a function of sex.

### 3.3. Student Satisfaction in Terms of Curricular Aspects

**Figure 2:**
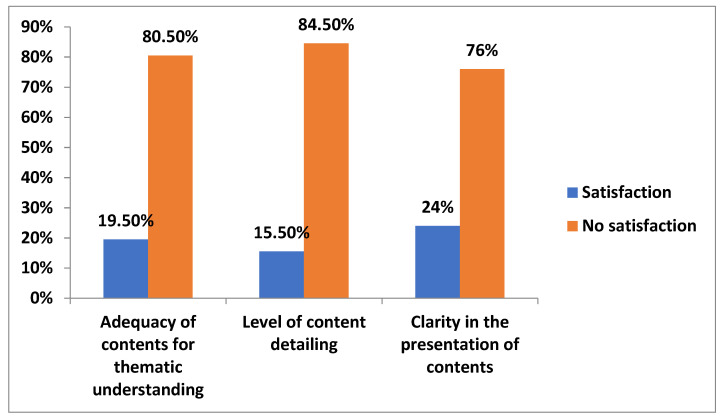
Student satisfaction with curricular aspects of virtual teaching.

A high percentage of students showed negative satisfaction with the curricular aspects, with the aspects related to the level of content detail presenting the worst results (84.50%), followed by the adequacy of content for thematic understanding (80.50%) and clarity in the presentation of content (76%) (Figure 2).

### 3.4. Student Satisfaction with Respect to the Didactic Proposal

**Figure 3:**
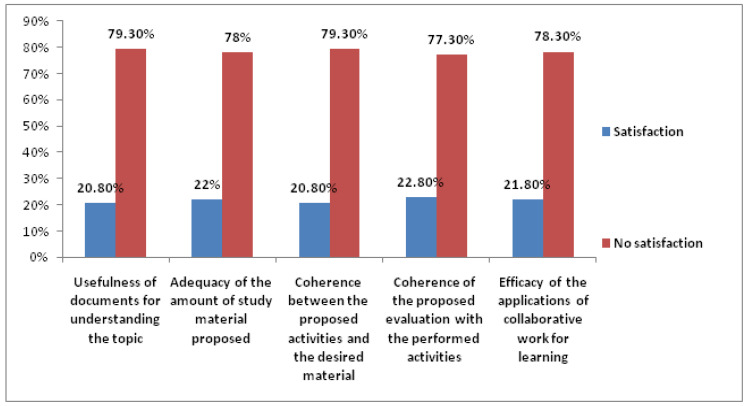
Student satisfaction with the didactic proposal in virtual teaching.

There was a considerably greater proportion of students dissatisfied with the didactic proposal carried out in virtual teaching than those satisfied with it (Figure 3).

### 3.5. Student Satisfaction in Terms of the Technological Environment and Tutoring

There was a greater percentage of dissatisfied students (72%) regarding the ease of access to the materials compared to those satisfied (28%).

With respect to the evaluation of the faculty response time, the percentages of satisfied and dissatisfied students were not very different. However, there were more students (57.3%) who were not satisfied with this aspect.

Regarding the clarity and relevance of the messages of the teacher (method, expectations and provided course materials/guidelines), a greater percentage of students presented dissatisfaction (74.8%) compared to those who were satisfied (25.3%).

Lastly, it is worth highlighting that not all students made enquiries with their faculties: 17% never did so, 65.3% claimed to have enquired sometimes, 15.3% did so frequently and 2.5% stated that they enquired always.

### 3.6. Relationship between Student Satisfaction in Virtual Teaching and Suffering from COVID-19, Sex and Realization of Face-to-Face Practical Activities

We conducted the Chi-squared independency test to determine whether the distribution of student satisfaction regarding curricular aspects was equal as a function of sex, suffering from COVID-19 and the realization of face-to-face practical activities. With a 95% confidence level, the results showed that there was no dependency among these variables (Table 1).

Regarding the analysis of the relationship between student satisfaction with the didactic proposals as a function of sex, suffering from COVID-19 and the realization of face-to-face practical activities, no dependency was observed between the variables (at a 95% confidence level, see Table 2).

Regarding the relationship between student satisfaction with respect to technological environment as a function of sex, suffering from COVID-19 and the realization of face-to-face practical activities, there was only dependency between the latter and student satisfaction (at a 95% confidence level, see Table 3). The percentage of students who did not carry out the face-to-face practical activities had a significantly greater percentage of dissatisfaction than those who did perform them.

Lastly, regarding the analysis of the relationship between student satisfaction with respect to tutoring as a function of sex, suffering from COVID-19 and the realisation of face-to-face practical activities, there was no dependency between these variables (at a 95% confidence level, see Table 4).

### 3.7. Relationship between Student Satisfaction with Virtual Teaching and Age

No statistically significant differences were observed in student satisfaction with respect to curricular aspects as a function of age (Student’s *t*-test: 0.284 (*p* = 0.777 in adequacy of content for thematic understanding); 0.394 (*p* = 0.694 in level of content detail); 0.210 (*p* = 0.834 in clarity in the presentation of content); at a 95% confidence interval).

In the analysis of the relationship of student satisfaction with the didactic proposal and age, there were no statistically significant differences (Student’s *t*-test: 0.080 (*p* = 0.936 in usefulness of documents for understanding the topic); −1.001 (*p* = 0.318 in adequacy of the amount of study material proposed); 0.291 (*p* = 0.771 in coherence between the proposed activities and desired material); −0.905 (*p* = 0.366 in coherence of the proposed evaluation with the performed activities); 1.447 (*p* = 0.149 in efficacy of the applications of collaborative work for learning); at a 95% confidence level).

Relative to student satisfaction regarding technological environment, there were no statistically significant differences as a function of age (at a 95% confidence level, see Table 5).

Lastly, no statistically significant differences were found in student satisfaction with tutoring as a function of age (Student’s *t*-test: −1.242 (*p* = 0.215 in faculty response time); −0.850 (*p* = 0.396 in clarity and relevance of the messages of the faculty); at a 95% confidence level).

## 4. Discussion

University-dependent careers such as nursing, medicine and veterinary medicine mainly base their teaching of the final year on practical activities that are relevant to clinical practice. With the COVID-19 pandemic, many countries had to adapt their educational programs to fully or partially online teaching [34]. This study aims to analyse the influence that this teaching methodology produced in the satisfaction of final year nursing students from different geographical areas.

Online practices were negatively received by some nursing students, possibly due to their perceptions of preparedness for entering the work force. According to some authors, face-to-face and contextualized practices consolidate the skills and abilities of observation, description and interpretation of one’s own context that will help introduce the necessary changes and transformations [35].

On the other hand, virtual teaching before the pandemic showed acceptable levels of student satisfaction. Most students were dissatisfied with the infrastructure [20,21]. However, the percentage of students dissatisfied with virtual teaching in this study has been high. This could be a consequence of a lack of planning [25]. In addition, access to resources (social, economic, health, etc.) during the pandemic could also have affected some people more than others. This pandemic has had greater impact on those of low socioeconomic status [36].

In an in-depth analysis of the curricular aspects of online teaching, a high dissatisfaction of the participants was observed in aspects such as the adequacy, clarity and level of the content. Regarding the didactic proposal and the tutoring of the teaching staff, most of the students showed dissatisfaction when comparing their experience with face-to-face teaching. Indeed, there were issues with the “coherence of the proposed evaluation with the activities carried out” [7,37], “coherence between the proposed activities and the desired material”, “adequacy of the amount of study material proposed”, “clarity and relevance of the teacher’s messages (method, expectations and provided course materials/guidelines)”, “access to materials”, etc. Research in this regard affirms that the teaching–learning process in the online teaching of careers such as medicine or nursing is less satisfactory for students than when teaching is face-to-face [7,14,37]. Student satisfaction is an indicator for assessing educational quality, so its immediate impact is directly related to motivation and performance. Additionally, to the extent that the expectations of students (designs, proactive teachers, efficient organization, technological equipment) are not met, the institution moves away from its goals. The imposition of digital educational culture brought fear and uncertainty. Frustration has been present in the perceptions of some students, as online education not only revealed existing educational inequalities, but added disadvantages, such as the limited guarantee of inclusion, quality and equity, bringing insecurity to students who perceived an undetermined future [25].

Finally, for the satisfaction variables studied previously, we analysed if there were differences depending on sex, age and whether the students had suffered from COVID-19 or not and if they had performed some practices in person, since some of the students did so. No differences were found in any of the cases.

Limitations of This Study

Despite the rise of novel topics related to the effects that the COVID pandemic has had on the training of different health professionals, such as the ones shown in this work, this study is not without its limitations. The main difficulty of this study is the lack of causality, since it is a collection of data from a self-reported questionnaire. In addition, this article only aims to give an overview of the objective of the study, and should be expanded with other analyses and a larger sample. Future research will turn in this direction.

In addition, this research has been conducted at a time when teachers had to resort to an “emergency” curriculum, which may not have been as effective as a well-thought-out and planned online curriculum, especially for courses that are more practical. Therefore, for a more comprehensive assessment of virtual teaching, we would have to study well-planned courses. It should be noted that the perception of students does not always coincide with the reality. A follow-up should be conducted in this population to assess if they still have this perception when they first work in the care sector.

## 5. Conclusions

The Degree of Nursing is a degree with an eminently practical character in every subject and with pre-professional practical activities. Such activities are taught in the form of independent clinical rotation training with a final evaluation of competencies in healthcare centres or hospitals that allow incorporating professional values, healthcare communication skills, clinical reasoning, clinical management and critical judgment. During the final year of the nursing curriculum, students are expected to integrate knowledge, skills and approaches related to professional practice. These specific skills are based on the principles and values associated with the competencies described in the general objectives and subjects that make up the Degree of Nursing (Specific competenciesE40. Degree Report, http://uhu.es/enfe/).

The COVID-19 pandemic has had a negative effect on the training of nursing students, specifically on those who were in the final year of the degree, as they could not carry out all the practical activities that would prepare them to enter the work force. Therefore, the present study is of great importance, as it offers the opportunity to value the satisfaction of the students of the Degree of Nursing with virtual teaching and use the results to improve different aspects of teaching, thus reducing the issues that were found during this period.

Most of the students of the Degree of Nursing had a negative experience with virtual teaching during the pandemic. This finding was analysed here through the satisfaction study of different academic parameters. However, the survey data were collected during a severe period of the pandemic, implying that other external variables may have influenced students’ perceptions of their learning. Therefore, it would be appropriate in future research to include elements in the data collection questionnaires on access to resources (social, economic, health, etc.) during the pandemic. This pandemic has left its mark on those at low socio-economic levels [4].

Teachers and students suffered the stresses of the pandemic [37,38]. It was a new situation for both groups. Therefore, it would be timely to implement mitigation strategies in pandemic situations. Since resources may be particularly scarce during these states, timely psychological support could also take many forms, such as telemedicine and informal support groups [38].

Among the most important aspects that were evaluated, it is important to highlight the lack of realization of practical activities and the deficit of training with the new COVID-19 disease.

Lastly, it is worth underlining that the percentages of dissatisfaction with the education received were even higher among the students who did not carry out face-to-face practical activities compared to those who did.

## Figures and Tables

**Table 1:** Group statistics and Pearson’s Chi-squared test for the variables of student satisfaction regarding curricular aspects as a function of sex, suffering from COVID-19 and the realization of face-to-face practical activities.

	Sex				Suffering from COVID-19				Realisation of Face-to-Face Practical Activities			
Curricular Aspects ^a^	Woman		Man		Yes		No		Yes		No	
Adequacy of contents for thematic understanding	Satisfaction (%)				Satisfaction (%)				Satisfaction (%)			
	No	Yes	No	Yes	No	Yes	No	Yes	No	Yes	No	Yes
	80.7	19.3	79.7	20.3	78.7	21.3	80.8	19.2	80.2	19.8	82.1	17.9
Pearson’s Chi-squared	0.033				0.142				0.130			
Asymptotic significance (bilateral)	0.856 *				0.706 *				0.719 *			
Level of content detailing	Satisfaction (%)				Satisfaction (%)				Satisfaction (%)			
	No	Yes	No	Yes	No	Yes	No	Yes	No	Yes	No	Yes
	84.9	15.1	82.6	17.4	80.3	19.7	85.2	14.8	84.1	15.9	86.6	13.4
Pearson’s Chi-squared	0.228				0.937				0.263			
Asymptotic significance (bilateral)	0.633 *				0.333 *				0.608 *			
Clarity in the presentation of contents	Satisfaction (%)				Satisfaction (%)				Satisfaction (%)			
	No	Yes	No	Yes	No	Yes	No	Yes	No	Yes	No	Yes
	75.8	24.2	76.8	23.2	77	23	75.7	24.3	75.4	24.6	79.1	20.9
Pearson’s Chi-squared	0.030				0.048				0.425			
Asymptotic significance (bilateral)	0.862 *				0.826 *				0.514 *			

^a^ Grouping variable: student satisfaction (Yes/No); * *p*-value of the Chi-squared test.

**Table 2:** Group statistics and Pearson’s Chi-squared test for the variables student satisfaction with the didactic proposal as a function of the variables sex, suffering from COVID-19 and the realisation of face-to-face practical activities.

	Sex				Suffering from COVID-19				Realisation of Face-to-Face Practical Activities			
Didactic Proposal ^a^	Woman		Man		Yes		No		Yes		No	
Usefulness of documents for understanding the topic	Satisfaction (%)				Satisfaction (%)				Satisfaction (%)			
	No	Yes	No	Yes	No	Yes	No	Yes	No	Yes	No	Yes
	81	19	71	29	77	23	79.6	20.4	78.7	21.3	82.1	17.9
Pearson’s Chi-squared	3.439				0.202				0.395			
Asymptotic significance (bilateral)	0.064 *				0.653 *				0.530 *			
Adequacy of the amount of study material proposed	Satisfaction (%)				Satisfaction (%)				Satisfaction (%)			
	No	Yes	No	Yes	No	Yes	No	Yes	No	Yes	No	Yes
	79.8	20.2	69.6	30.4	78.7	21.3	77.8	22.2	78.1	21.9	77.6	22.4
Pearson’s Chi-squared	3.457				0.023				0.007			
Asymptotic significance (bilateral)	0.063 *				0.879 *				0.993 *			
Coherence between the proposed activities and desired material	Satisfaction (%)				Satisfaction (%)				Satisfaction (%)			
	No	Yes	No	Yes	No	Yes	No	Yes	No	Yes	No	Yes
	78.5	21.5	82.6	17.4	80.3	19.7	79	21	79.3	20.7	79.1	20.9
Pearson’s Chi-squared	0.572				0.056				0.001			
Asymptotic significance (bilateral)	0.449 *				0.813 *				0.974 *			
Coherence of the proposed evaluation with the performed activities	Satisfaction (%)				Satisfaction (%)				Satisfaction (%)			
	No	Yes	No	Yes	No	Yes	No	Yes	No	Yes	No	Yes
	77.6	22.4	75.4	24.6	75.4	24.6	77.5	22.5	76.3	23.7	82.1	17.9

^a^ Grouping variable: student satisfaction (Yes/No); * *p*-value of the Chi-squared test.

**Table 3:** Group statistics and Pearson’s Chi-squared test for student satisfaction with respect to technological environment as a function of sex, suffering from COVID-19 and the realisation of face-to-face practical activities.

	Sex				Suffering from COVID-19				Realisation of Face-to-Face Practical Activities			
Technological Environment ^a^	Woman		Man		Yes		No		Yes		No	
Ease of access to the different materials	Satisfaction				Satisfaction				Satisfaction			
	No	Yes	No	Yes	No	Yes	No	Yes	No	Yes	No	Yes
	73.4	26.6	65.2	34.8	65.6	34.4	73.1	26.9	70	30	82.1	17.9
Pearson’s Chi-squared	1.903				1.441				4.064			
Asymptotic significance (bilateral)	0.168 *				0.230 *				0.044 *			

^a^ Grouping variable: student satisfaction (Yes/No); * *p*-value of the Chi-squared test.

**Table 4:** Group statistics and Pearson’s Chi-Squared test for student satisfaction with respect to tutoring as a function of sex, suffering from COVID-19 and the realisation of face-to-face practical activities.

	Sex				Suffering from COVID-19				Realisation of Face-to-Face Practical Activities			
Tutoring ^a^	Woman		Man		Yes		No		Yes		No	
Faculty response time	Satisfaction (%)				Satisfaction (%)				Satisfaction (%)			
	No	Yes	No	Yes	No	Yes	No	Yes	No	Yes	No	Yes
	56.8	43.2	59.4	40.6	60.7	39.3	56.5	43.5	56.8	43.2	59.7	40.3
Chi-Cuadrado De Pearson	0.160				0.363				0.198			
Asymptotic significance (bilateral)	0.689 *				0.547 *				0.657 *			
Clarity and relevance of the messages of the faculty	Satisfaction (%)				Satisfaction (%)				Satisfaction (%)			
	No	Yes	No	Yes	No	Yes	No	Yes	No	Yes	No	Yes
	75.5	24.5	71	29	77	23	74.3	25.7	74.2	25.8	77.6	22.4
Pearson’s Chi-squared	0.616				0.213				0.349			
Asymptotic significance (bilateral)	0.432 *				0.645 *				0.555 *			

^a^ Grouping variable: student satisfaction (Yes/No); * *p*-value of the Chi-squared test.

**Table 5:** Group statistics and Student’s *t*-test for student satisfaction regarding technological environment and age.

	Age	
Technological Environment ^a^	X^−^	
	No	Yes
Ease of access to the different materials	23.38	23.04
Student’s *t*-test	0.759	
Asymptotic significance (bilateral)	0.448 *	

^a^ Grouping variable: student satisfaction (Yes/No); * *p*-value of the Student’s *t*-test.

## Data Availability

The data that support the findings of this study are available from the corresponding author upon reasonable request.

## Supplementary Materials

The following supporting information can be downloaded at: https://www.mdpi.com/article/10.3390/ijerph19042023/s1, Table S1: Distribution of the sample by educational institution.
