# Supplementary material for: Nursing Education during the SARS-CoV-2 Pandemic: Assessment of Students’ Satisfaction with e-Learning Environment
Source: Int J Environ Res Public Health. 2022 Feb 11;19(4):2023. doi: 10.3390/ijerph19042023 (PMC8872368; doi:10.3390/ijerph19042023)
Supplement: Supplementary file 1 [file ijerph-19-02023-s001.zip › ijerph-1503397-supplementary.pdf]

Table S1. Distribution of the sample by educational institution.

University Center	N	%
Centro Universitario de Enfermería "San Juan de Dios"	4	1
Centro Universitario de Enfermería de Cruz Roja	17	4.3
CEU San Pablo	1	0.3
Escuela de Enfermería Salus Infirmorum	11	2.8
Escuela Universitaria de Osuna	7	1.8
EUE Gijón	1	0.3
Europea	1	0.3
Fundación Jiménez Díaz	5	1.3
UAH	1	0.3
Ucav	1	0.3
Universidad Autónoma de Madrid	4	1
Universidad Católica de Ávila	3	0.8
Universidad Católica de Murcia	13	3.3
Universidad Complutense de Madrid	2	0.5
Universidad de Alcalá de Henares	1	0.3
Universidad de Alicante	1	0.3
Universidad de Burgos	3	0.8
Universidad de Cádiz	42	10.5
Universidad de Cantabria	1	0.3
Universidad de Castilla la Mancha	7	1.8
Universidad de Chile	5	1.3
Universidad de Córdoba	15	3.8
Universidad de Extremadura	9	2.3
Universidad de Granada	9	2.3
Universidad de Huelva	122	30.6
Universidad de Jaén	11	2.8
Universidad de las Américas	1	0.3
Universidad de las Islas Baleares	1	0.3
Universidad de las Palmas	4	1
Universidad de León	2	0.5
Universidad de Lleida	3	0.8
Universidad de Málaga	1	0.3
Universidad de Murcia	4	1
Universidad de Navarra	8	2
Universidad de Osuna	1	0.3
Universidad de Oviedo	13	3.3
Universidad de Oviedo (Facultad de Enfermería de Gijón)	1	0.3

Universidad de Plasencia	1	0.3
Universidad de Salamanca	11	2.8
Universidad de Salamanca (USAL)	1	0.3
Universidad de Santiago de Compostela	6	1.5
Universidad de Sevilla	16	4
Universidad de Valencia	1	0.3
Universidad de Vigo	1	0.3
Universidad de Zaragoza	1	0.3
Universidad Europea de Madrid	1	0.3
Universidad Francisco de Vitoria	14	3.5
Universidad Pompeu Fabra	1	0.3
Universidad Pontificia de Salamanca	1	0.3
Universidad pública del País Vasco	1	0.3
Universidad de Chaves	1	0.3
Universitat de Barcelona	3	0.8
Universitat de Lleida	1	0.3
Universitat Rovira i Virgili	3	0.8
Total	400	100
